# A Microflow Cytometer with a Rectangular Quasi-Flat-Top Laser Spot

**DOI:** 10.3390/s16091474

**Published:** 2016-09-11

**Authors:** Jingjing Zhao, Zheng You

**Affiliations:** 1 State Key Laboratory of Precision Measurement Technology and Instrument, Tsinghua University, Beijing 100084, China; jing.jing.youxiang@163.com; 2 Department of Precision Instrument, Tsinghua University, Beijing 100084, China; 3 Beijing Laboratory for Biomedical Detection Technology and Instrument, Tsinghua University, Beijing 100084, China

**Keywords:** three dimensional hydrodynamic focusing, microfluidics, binary optical element, microflow cytometer

## Abstract

This work develops a microflow cytometer, based on a microfluidic chip for three-dimensional (3D) hydrodynamic focusing and a binary optical element (BOE) for shaping and homogenizing a laser beam. The microfluidic chip utilizes sheath flows to confine the sample flow along the channel centerline with a narrow cross section. In addition to hydrodynamic focusing, secondary flows are generated to strengthen the focusing in the vertical direction. In experiments, the chip was able to focus the sample flow with cross sections of 15 μm high and 8–30 μm wide at 5 m/s, under the condition of the sample flow rates between 10 and 120 μL/min. Instead of using the conventional elliptical Gaussian spot for optical detection, we used a specially designed BOE and obtained a 50 μm × 10 μm rectangular quasi-flat-top spot. The microflow cytometer combining the chip and the BOE was tested to count 3, 5, and 7 μm fluorescence microbeads, and the experimental results were comparable to or better than those derived from two commercial instruments.

## 1. Introduction

Recent advances in microfluidic technologies have brought about developments in microflow cytometers. Among these technologies, 3D focusing is essential for improving throughput, stability, and sensitivity. A number of focusing methods based on different physical mechanisms have been developed, mainly including electrokinetic focusing [1,2,3], acoustic focusing [4,5], inertial focusing [6,7,8,9,10], microstructure-induced secondary-flow focusing [11,12,13,14,15], and hydrodynamic focusing, among which hydrodynamic focusing can operate over a wide velocity range and has no special demands for particles or fluidic mediums, as demonstrated in conventional flow cytometers [16]. To achieve 3D hydrodynamic focusing in a microfluidic chip, usually there is a need to manipulate multiple sheath flows independently to envelope and squeeze the sample flow in vertical and horizontal directions [17,18,19,20,21,22], followed with complicated microfluidic manifolds, flow control, and declining performance. To overcome these challenges, this paper establishes a 3D hydrodynamic focusing design in a microfluidic chip, comprising two sheath-flow channels and one sample-flow channel, where horizontal and vertical focusing are implemented successively. In operation, the two sheath-flow channels are connected to the same sheath flow source, thus we only need to control one sheath flow and one sample flow. To further improve the 3D focusing, secondary flows are introduced by microstructures to vertically strengthen the focusing, and the focusing performance is comparable to conventional flow cytometers.

Laser beam shaping is another fundamental technology for detection. Conventional flow cytometers use elliptical focal spots that are 5–20 μm high and 100 μm wide, which are provided by two crossed cylindrical lenses with different focal lengths [23]. For microflow cytometers, circular spots of tens of micrometers are commonly used, which can be easily shaped by an objective [24,25]. Elliptical spots are also applied, which are obtained by an objective lens with a slit [26,27,28] or a cylindrical lens [29,30]. Apart from bulk optical elements, on-chip waveguides, fibers, and lenses can build a compact and easy-to-use illumination and detection system [31,32,33,34], but the focused spot provided by on-chip elements is relatively large, which severely limits the spatial resolution [35] and the throughput. All of the above spots have Gaussian intensity distributions, and their tops are not flat and their boundaries are not sharp or straight. Therefore, the fluorescence signal from a cell excited by the spot will depend on its route passing through the spot. Ideally, the laser spot should be rectangular and have a flat-top. To achieve this goal, a binary optical element (BOE) is designed to shape and homogenize the laser beam, resulting in a 50 μm × 10 μm quasi-flat-top spot. The BOE and focusing chip are combined to work as a microflow cytometer. It was experimentally tested by counting fluorescence microbeads, giving similar or better results in comparison with two commercial instruments, BD Accuri C6 and BD LSR Fortessa.

This work develops a microfluidic chip, a BOE, and a microflow cytometer. The focusing chip provides comparable focusing performance to conventional flow cytometers, specifically in terms of high sample flow rate, high flow velocity, and small cross-sectional dimensions of the focused sample flow. It can be used for microflow cytometry [17,18,19,20,21,22], and potentially for other microfluidic systems where hydrodynamic focusing is needed, such as the synthesis of polymeric particles [36], nanoparticles for drug delivery [37], or DNA compaction for gene therapy [38]. Based on the principles of optical diffraction, the BOE is capable of generating a rectangular quasi-flat-top spot. It explores the possibility of utilizing BOEs to achieve the spots with special shapes and energy distributions, which cannot be generated by geometrical optics. Thus, researchers are able to design and produce a desired spot to meet the demands of the illumination in a microfluidic system. A powerful microflow cytometer has been established based on this work which is competitive with conventional flow cytometers.

## 2. Design

### 2.1. Microfluidic Chip for 3D Hydrodynamic Focusing

**Figure 1:**
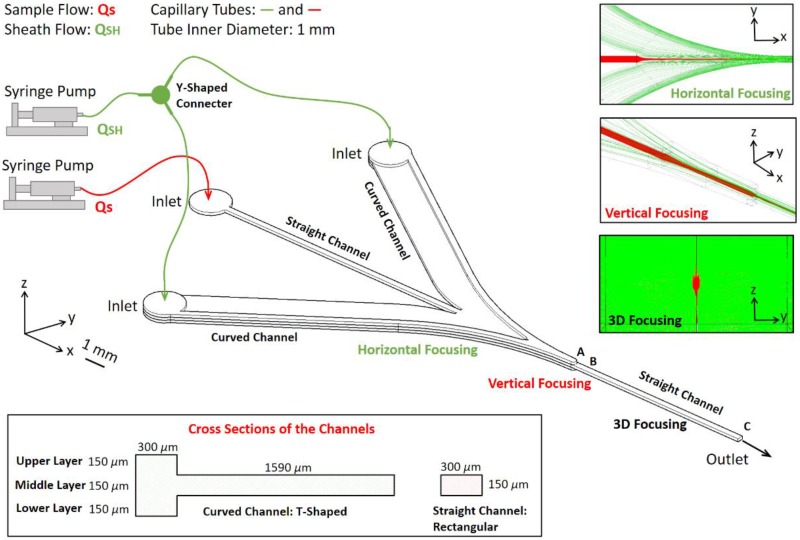
Schematic diagram of the 3D focusing microfluidic chip.

The microfluidic chip is comprised of three microchannel layers, as illustrated in Figure 1. There are two symmetrical curved channels smoothly merging into the straight sample-flow channel. The design process for the curved channels is expressed minutely in our previous work [39]. The cross sections of the two curved channels are T-shaped, therefore the sheath fluids in the middle layer first accomplish horizontal focusing by pressuring the sample low from the right and left sides, and then the fluids in the upper and lower layers cover the sample flow from above and below to further achieve vertical focusing. Near the ends of the channels in the upper and lower layers (position A in Figure 1), the sheath fluids are forced into the straight channel in the middle layer to perform hydrodynamic focusing completely. Two syringe pumps are applied to manipulate the flow rates of the sample flow and the sheath flow respectively, and the pumps are connected to the microchannel inlets using capillary tubes. The sheath flow streams into the two curved channels are identically the same through an off-chip Y-shaped connecter.

**Figure 2:**
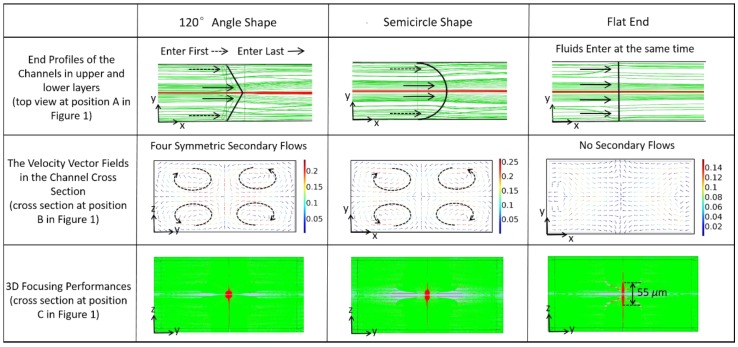
When the end profiles of the channels in the upper and lower layers are designed as convex shapes, secondary flows will intensify the vertical focusing in the straight channel. The simulated results were obtained at *Q_S_* = 60 μL/min and *Q_SH_* = 7.2 mL/min.

The channel end profiles of the channels in the upper and lower layers can influence the focusing performance. When the end profiles are flat, all the sheath fluids enter the straight channel in the middle layer at the same time. In consequence, the height of the sample flow is only reduced by two thirds, which is consistent with the decrease in channel height (the three microchannel layers have the same height). To reinforce the vertical focusing, the end profile is designed as a convex shape. Thus, the sheath fluids close to the right and left channel walls firstly go into the straight channel and the sheath fluids near the horizontal centerline enter last. Since the fluids near the channel center have higher velocities than the fluids near the walls (the velocity profile in the channel is parabolic), they have greater momentum, resulting in four symmetrical secondary flows in the straight channel, which significantly intensify vertical focusing and maintain the sample flow at the center position. As convex shapes, semicircle shapes, and angle shapes are specifically investigated for the end profile through simulations by COMSOL software, it is found that these shapes can further decrease the sample flow height and the 120° angle shape exhibits the best performance. Additionally, the vertical focusing strength increases with increasing flow velocity or sheath flow rate. As the fluids pass the constriction position (Position A in Figure 1), the secondary flows will lose momentum and gradually vanish, and the cross-sectional profile of the focused sample flow will reach a stable state. As observed both in simulations and experiments, the distance for the stabilization of fluids is no more than 1 mm in all the cases studied in this paper. The above analysis is presented in Figure 2 and will be further discussed in the experimental section. For fabrication convenience, the semicircle-shaped end profile is applied in our design.

### 2.2. BOE for Laser Beam Shaping and Homogenization

**Figure 3:**
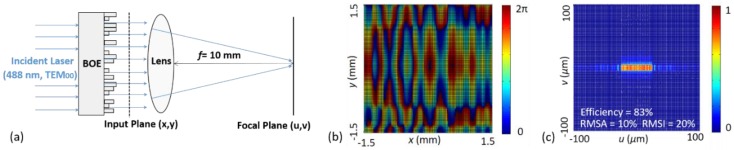
The binary optical element (BOE) for beam shaping and homogenization: (**a**) the illumination system; (**b**) the discretized phase profile of the BOE; (**c**) the intensity distribution on the focal plane.

BOE is a stepped surface-relief optical element, which retards the incident light by a modification of the surface profile [40]. Thus, the light emitted from different zones of the BOE interferes and forms the desired wavefront. Using a specially designed BOE, the Gaussian profile of the laser beam can be transformed into a rectangle with a quasi-uniform intensity distribution, which is preferable for flow cytometry. The illumination system based on BOE is shown in Figure 3a, which is essentially a Fourier transformation system. The incident laser *G_0_*(*x*,*y*) can be represented by *A*(*x*,*y*)exp[iΦ_0_(*x*,*y*)], where *A*(*x*,*y*) represents the amplitude and Φ_0_(*x*,*y*) the phase of the light field. Since the laser is single model, Φ_0_(*x*,*y*) is constant. After the laser has passed through the BOE, the output wave *G*(*x*,*y*) is described by *A*(*x,y*)exp[iΦ*_0_*(*x,y*) + iΦ*_B_*(*x,y*)], where Φ*_B_*(*x*,*y*) is the phase deformation introduced by the BOE. The relationship between Φ_B_(*x*,*y*) and the BOE surface profile *h*(*x*,*y*) is given by

(1) $$\Phi_{B}\left(x,y\right)=\left(2\pi /\lambda \right)\left(n-1\right)h\left(x,y\right)$$

where *n* is the BOE refractive index and *λ* is the laser wavelength. Then, the wavefront *G*(*x*,*y*) converges to form the desirable spot on the focal plane of the lens. The light filed on the focal plane is *U*(*u*,*v*). The Fourier transformation (FT) from *G*(*x*,*y*) to *U*(*u*,*v*) is given below

(2) $$\left\{ \begin{array}{l} U\left(u,v\right)=\frac{1}{\lambda f}\iint G\left(x,y\right)\exp\left[-i2\pi \left(xf_{x}+yf_{y}\right)\right]dxdy=\frac{1}{\lambda f}\mathrm{FT}\left[G\left(x,y\right)\right]\\ \left(f_{x},f_{y}\right)=\left(\frac{u}{\lambda f},\frac{v}{\lambda f}\right) \end{array}\right.$$

where (*f_x_*, *f_y_*) represents spatial frequency and *f* is the focal length of the lens. The BOE is a square with a side-length *D*, and its surface is divided into *N_s_* × *N_s_* zones, where the side-length is *D*/*N_s_*. According to Fourier optics, the area of *U*(*u*,*v*) on the focal plane is (*N_s_*·Δ)^2^, here Δ = *λf*/*D* is the sampling interval. The relationships between *G*(*x*,*y*) and *U*(*u*,*v*) are further expressed by fast Fourier transformation (FFT)

(3) $$\left\{ \begin{array}{l} U\left(u,v\right)=\frac{1}{\lambda f}dXdY\sum_{y=-dY\cdot N_{S}/2}^{dY\left(N_{S}/2+1\right)}\sum_{x=-dX\cdot N_{S}/2}^{dX\left(N_{S}/2+1\right)}G\left(x,y\right)\exp\left[-i2\pi \left(ux+vy\right)\right]=\frac{1}{\lambda f}dXdY\cdot \mathrm{FFT}\left[G\left(n_{x},n_{y}\right)\right]\\ dX=dY=D/N_{S} \end{array}\right.$$

(4) $$\left\{ \begin{array}{l} G\left(x,y\right)=\lambda f\sum_{v=-dV\cdot N_{S}/2}^{dV\left(N_{S}/2+1\right)}\sum_{u=-dU\cdot N_{S}/2}^{dU\left(N_{S}/2+1\right)}U\left(u,v\right)\exp\left[i2\pi \left(ux+vy\right)\right]=\lambda f\cdot {\mathrm{FFT}}^{-1}\left[U\left(n_{u},n_{v}\right)\right]\\ dU=dV=\lambda f/D \end{array}\right.$$

which can be processed by a computer. In this work, the laser is 488 nm (TEM_00_) with a diameter of 3 mm, the BOE side-length is 3 mm, the lens focal length is 10 mm, and the sampling number *N_s_* is 128. The desired spot on the focal plane is a 50 μm × 10 μm rectangle with an absolutely flat top. Using a modified GS algorithm [41,42], an iterative algorithm, the optimal solution of Φ*_B_*(*x,y*) is found. Since the BOE is fabricated using very large scale integration (VLSI) technologies, 2^4^ surfaces can be generated through photolithography and etching processes when using four masks. Naturally, the optimal solution of Φ*_B_*(*x,y*) needs discretizing to 2^4^ phase levels and the interval *dP* is π/8, as calculated below.

(5) $${\bar{\Phi }}_{B}\left(x,y\right)=\left\{ \begin{array}{ll} \mathrm{round}\left[\Phi_{B}\left(x,y\right)/dP\right]\cdot dP & \mathrm{round}\left[\Phi_{B}\left(x,y\right)/dP\right]\neq 0\\ \;dP & \;\mathrm{round}\left[\Phi_{B}\left(x,y\right)/dP\right]=0 \end{array}\right.$$

The discretized solution $\bar{\Phi }$_B_(*x*,*y*) is shown in Figure 3b and the intensity distribution generated by $\bar{\Phi }$_B_(*x*,*y*) is demonstrated in Figure 3c. A 50.4 μm × 9.8 μm rectangular spot is successfully obtained, whose boundary is very sharp. This spot contains more than 83% of the incident laser energy, and the relative root mean square errors of amplitude and intensity (RMSA and RMSI) are 10% and 20%, respectively. The efficiency *η*, RMSA, and RMSI are given by

(6) $$\eta =\frac{\sum \sum U_{SP}{\left(u,v\right)}^{2}dUdV}{\sum \sum A{\left(x,y\right)}^{2}dXdY}$$

(7) $$\left\{ \begin{array}{l} RMSA=\sqrt{\frac{\sum \sum {\left[\left|U_{SP}\left(u,v\right)\right|/A_{MEAN}-1\right]}^{2}}{N_{SP}-1}}\\ A_{MEAN}=\frac{\sum \sum \left|U_{SP}\left(u,v\right)\right|}{N_{SP}} \end{array}\right.$$

(8) $$\left\{ \begin{array}{l} RMSI=\sqrt{\frac{\sum \sum {\left[U_{SP}{\left(u,v\right)}^{2}/I_{MEAN}-1\right]}^{2}}{N_{Z}-1}}\\ I_{MEAN}=\frac{\sum \sum U_{SP}{\left(u,v\right)}^{2}}{N_{SP}} \end{array}\right.$$

where *U_SP_*(*u*,*v*) is the spot region and *N_SP_* is the number of the sampling points in this region. The top is quasi-flat, not perfectly uniform, which is also a common phenomenon in other BOE homogenizers. The dimensions of the spot are 50.4 μm × 9.8 μm which is very close to the desired spot size, 50 μm × 10 μm.

## 3. Experimental Setup

### 3.1. Microfluidic Chip

**Figure 4:**
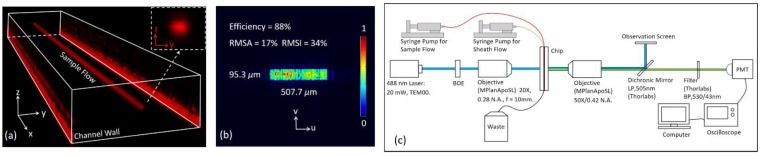
(**a**) The focused sample flow was a narrow stream along the centerline in the straight channel; (**b**) The spot intensity distribution captured by the profiler; (**c**) The microflow cytometer schematic.

The microfluidic chip was fabricated to experimentally characterize the 3D hydrodynamic focusing. A chip was constructed by five glass plates, where microstructures were carved by precision machining. The inner three 150 μm height plates contained the microchannels, and two 1 mm and 2 mm plates on top and bottom respectively covered the inner structure for optical observation, fluid inlet and outlet ports, and protection. The five glass plates were integrated via UV adhesive bonding. Figure S1 illustrates the microfluidic chip. Two syringe pumps (TS-1B, LongerPump Inc., Baoding, China) were manipulated to drive the sample flow and the sheath flow respectively. The sample flow was rhodamine B aqueous solution (red), and the sheath flow was sodium fluorescein aqueous solution (green) or just distilled water. Taking advantage of confocal imaging (captured by Nikon A1), the focusing characteristics were visualized in Figure 4a. According to fluorescence intensity distribution, the cross-sectional profiles of the focused sample flow were quantified by the full width at half-maximum (FWHM) algorithm [20,43], and the measuring process is illustrated in Figure S2.

### 3.2. BOE

The BOE was built on a quartz wafer through VLSI fabrication techniques, using four masks to generate 2^4^ phase levels. Part of the BOE was displayed in Figure S3. The spot generated by the BOE was analyzed by a power meter (FieldMaxII, Coherent, Santa Clara, CA, USA) and a laser beam profiler (BC106N-VIS, Thorlabs, Newton, NJ, USA). The diffractive efficiency of the BOE was 88%, close to the predicted value, 83%. The pixel size of the profiler is 6.45 μm. In order to improve measurement precision, a lens of 100 mm focal length was used instead of 10 mm focal length, thus the spot size was magnified tenfold. Figure 4b presents the intensity distribution captured by the profiler. The rectangular spot was 504.5 μm × 99.7 μm. The spot homogeneity was not as good as expected, as the spot RMSA and RMSI were 17% and 34%, respectively, higher than the predictions. Generally, the homogeneity degradation was considered the result of fabrication errors. (A spatial light modulator can be used as a convenient alternative for BOE.)

### 3.3. Microflow Cytometer

Based on the focusing chip and the BOE, a microflow cytometer was established, as shown in Figure 4c and Figure S4. Fluorescence microbeads (Polystyrene Spheres, 488 nm/530 nm, Huge Biotech. Inc., Shanghai, China) of 3, 5, and 7 μm diameters were suspended in distilled water as the sample fluid, and the sheath fluid was distilled water. The concentration for 3 μm beads was 2.0 × 10^7^ particles/mL, 2.9 × 10^6^ particles/mL for 5 μm beads, and 1.6 × 10^6^ particles/mL for 7 μm beads. Microbeads were confined at the channel center and excited by the 50 μm × 10 μm rectangular spot, whose long side was perpendicular to the flow direction. Emitted fluorescence from beads was detected and linearly amplified by a photomultiplier tube (PMT, R928+C7427, Hamamatsu, Hamamatsu City, Japan). The PMT signals were recorded by an oscilloscope (MSO1104Z, 1 GSa/s, Rigol, Beijing, China) and further analyzed in Matlab.

## 4. Results and Discussions

### 4.1. Flow Configuration in Key Regions of the Microfluidic Chip

**Figure 5:**
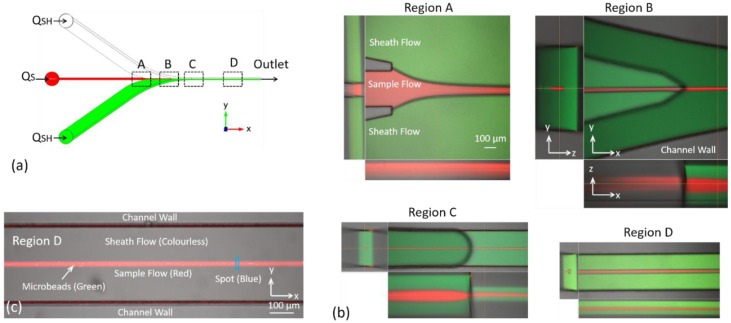
Flow configuration in key regions: (**a**) the key regions in the design and (**b**) the confocal images where the sample flow was red and sheath flow was green. The images were obtained under the condition of *Q_S_* = 60 μL/min, *Q_SH_* = 7.2 mL/min, and *V_S_* = 5.2 m/s. (**c**) The red sample flow contained 3 μm green microbeads, and the sheath flow was distilled water with no fluorescein.

To visually and fully explain the principles of the 3D hydrodynamic focusing achieved in the chip, the confocal images of the key regions were captured and shown in Figure 5. Horizontal focusing first happened in Region A and in Region B the sample flow was completely encircled by sheath fluids. Then in Region C vertical focusing worked and the final performance was tested in Region D, where 3D hydrodynamic focusing reached a steady state. In all the experiments investigated, the focused sample flow was at the center position with deviations of a few microns. Figure 5c indicates the position of the rectangular spot, whose long side is perpendicular to the flow direction.

### 4.2. Characteristics of the Focusing Chip

**Figure 6:**
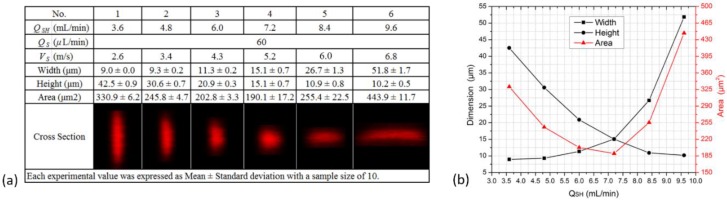
Experimental results of the focused sample flow when the sheath flow increased from 3.6 to 9.6 mL/min (or the sample flow velocity increased from 2.6 to 6.8 m/s), as shown in the table (**a**) and the figure (**b**).

**Figure 7:**
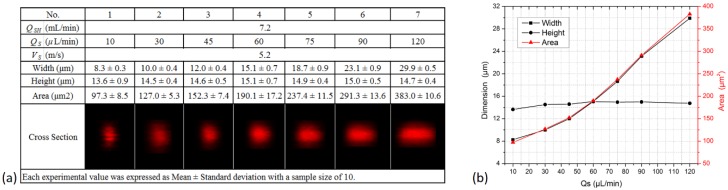
Experimental results of the focused sample flow when the chip operated at a constant velocity of 5.2 m/s and the sample flow rate increased from 10 to 120 μL/min, as shown in the table (**a**) and the figure (**b**).

The chip utilizes secondary flows, which are sensitive to flow velocity. Thus the optimal operating velocity for focusing should be found out for achieving the desired 3D focusing. In the experiments, the sheath flow rate *Q_SH_* was adjusted between 3.6 and 9.6 mL/min with a constant sample flow rate *Q_S_* of 60 μL/min, hence the velocity *V_S_* of the focused sample flow varied from 2.6 to 6.8 m/s (the velocity at the channel center was about two times the average velocity [44], thus *V_S_* = 2(*Q_SH_* + *Q_S_*)/*A_CS_* ≈ 2*Q_SH_*/*A_CS_*, where *A_CS_* is the cross-sectional area of the straight microchannel). The results are presented in Figure 6. All the cross sections were rounded and symmetric, resulting from the vertical focusing process assisted by the four equal and symmetric secondary flows. With increasing flow velocity, the focused sample flow changed from vertical ellipses to circular shapes to horizontal ellipses. It was at 5.2 m/s that the sample flow had a circular cross section with a diameter of 15 μm and the area was the smallest. In this case, 5.2 m/s was determined as the optimal velocity. In practice, a flow cytometer should be able to effectivly work when the sample flow rate changes and the sheath flow rate keeps constant. The design was tested with *Q_S_* varying from 10 to 120 μL/min and *Q_SH_* being fixed at 7.2 mL/min, and the optimal velocity was achieved. The results are presented in Figure 7. It shows that the height of the sample flow was almost constant and barely affected by the sample flow rate, while the width and the area were both proportional to the rate. This relationship between dimensions and rate was also observed in simulations. As sample flow rate increased from 10 to 120 μL/min, the height kept around 15 μm and the width rose from 8 to 30 μm. In addition, the standard deviation of every measurement was quite small relative to its mean value, demonstrating good stability and repeatability of the focusing chip. We further compared the design with conventional flow cytometers. Taking BD Calibur and BD Accuri C6, for example, the former operates at roughly 6 m/s with the sample flow rate of 12–60 μL/min and a focused diameter of 6.5–15 μm, the latter works at about 3 m/s with a flow rate of 14–66 μL/min and a focused diameter of 10–22 μm. Obviously, the design has achieved similar favorable focusing performances with the two instruments, in terms of sample flow velocity, sample flow rate, and the focused cross-sectional dimensions.

### 4.3. Characteristics of the Fluorescence Signals

**Figure 8:**
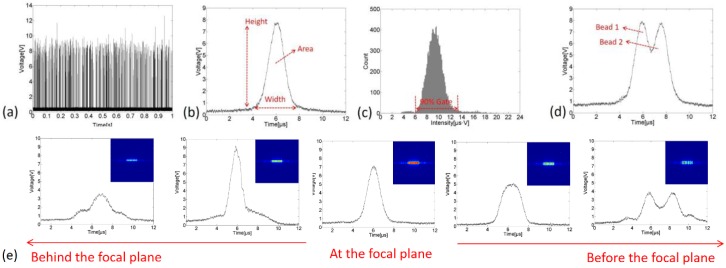
Fluorescence signals captured by the microflow cytometer system, (**a**) signals emitted from 5 μm beads during 1 s time windows, (**b**) signal waveform of a 5 μm bead, (**c**) a typical intensity histogram of 5 μm beads, (**d**) the adhesion of two beads could be recognized according to its fluorescence signal, (**e**) the signal waveform deformed when the sample flow was before or behind the focal plane, with the corresponding light intensity distributions shown in insert graphs, and it shows that at the focal plane the spot displayed the best homogeneity and most accurate dimensions.

The micrflow cytometer was operated to count microbeads at the sample flow rate of 60 μL/min and the sheath flow rate of 7.2 mL/min. The fluorescence signals emitted from beads during 1 s time windows were shown in Figure 8a. The throughputs of 3, 5, and 7 μm were 20,000 events per second (eps), 2300 eps, and 1400 eps, respectively. Figure 8b shows the signal waveform of one bead, and the pulse area could represent the signal intensity. The pulse width was about 3–4 μs. It could be expected that the throughput can achieve as high as 100 k eps if on average one bead passes through the spot every 10 μs. Figure 8c shows an intensity histogram of 12,000 beads, and there is a 90-percent-gate used to exclude the samples drastically deviating from the average. The coefficient of variation (CV) of the samples inside the gate was calculated in order to evaluate the stability of this system, which is a key indicator for flow cytometer. In addition, the spot short-side was 10 μm and it was easy to recognize the adhesion of two beads from the fluorescence signal, as shown in Figure 8d. In operation, the distance between the lens for Fourier transform and the chip needs adjusting so that the focused sample flow is at the focal plane of the lens. As presented in Figure 8e, the desired distance could be easily determined according to the signal waveform, since on the plane before or behind the focal plane the spot deformed and the waveform changed accordingly.

### 4.4. CVs of Microbeads under Different Sample Flow Rates

**Figure 9:**
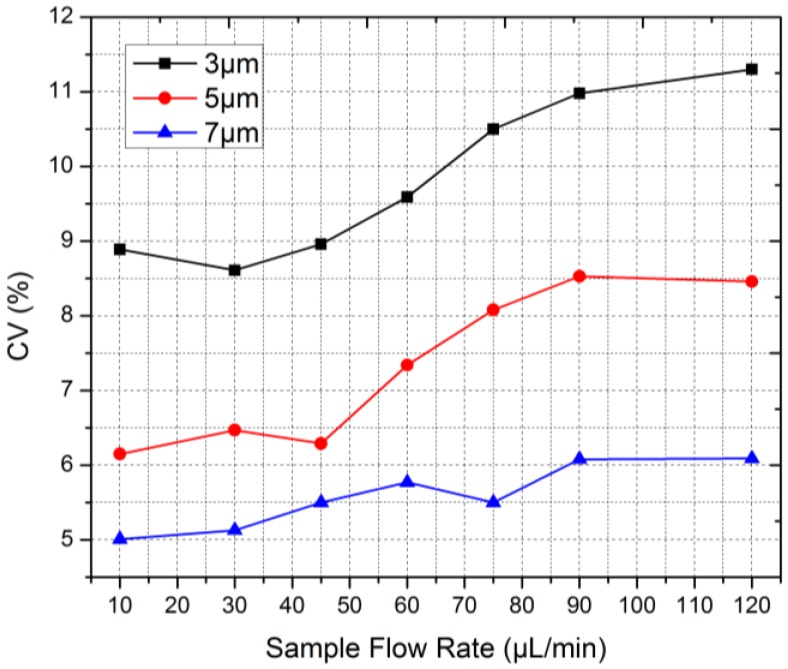
An analysis of 3, 5, and 7 μm microbeads was conducted by the microflow cytometer under different sample flow rates, and the sheath flow was kept at 7.2 mL/min to make the focusing chip operate at the optimal velocity.

According to the focusing experiments in Section 4.2, the sheath flow rate was set at 7.2 mL/min, thus the focusing chip worked at the optimal velocity, and the sample flow rate was increased from 10 to 120 μL/min. An analysis of 3, 5, and 7 μm beads was conducted and the results were depicted in Figure 9. For credibility, the sample size for every test was not less than 12,000. With the increasing sample flow rate, the CVs of the 3, 5, and 7 μm beads all got larger, indicating that the stability of the microflow cytometer decreased. This was because the spot was not absolutely uniform and the cross-sectional area of the focused sample flow increased with the sample flow rate, as shown in Figure 7, thereby expanding the distribution of beads in the channel cross section. Thus the differences of laser illumination among beads enlarged and the amplitude fluctuations of signals increased (Video S1), resulting in bigger CVs. Another observable phenomenon was that large beads went with small CVs while small beads with large CVs. There were two reasons. First, the distribution of large beads in the sample flow cross section was relatively more concentrated when compared with that of small beads. Second, large beads had more mass and inertia, which contributed to them maintaining themselves at the central position of the channel cross section. Those two factors could reduce the illumination difference.

The samples were also analyzed by two commercial flow cytometer, BD Accuri C6 and BD LSR Fortessa, and the commercial instruments ran at low, medium, and high sample flow rates. The results were listed in Table 1. For all the tests, the microflow cytometer was better than C6 with smaller CVs. Compared with Fortessa, the microflow cytometer was inferior when counting 3 μm beads but superior when counting 7 μm beads. For 5 μm beads, the micro and Fortessa provided nearly equal performances. It showed that the microflow cytometer was better at microbeads with large diameters. In conclusion, the microflow cytometer presented a strong competitive performance.

## 5. Conclusions

This work develops a microflow cytometer formed by integrating a microfluidic chip for 3D hydrodynamic focusing and a BOE for beam shaping and homogenization. The chip combines the hydrodynamic method and secondary flows to accomplish 3D focusing, which is capable of confining the sample flow with the width and height dimensions of 10–30 μm at 5 m/s when the sample flow rate varies between 10 and 120 μL/min. The BOE is based on Fourier optics and was designed by a modified GS algorithm, and it can generate a 50 μm × 10 μm rectangular quasi-flat-top spot for illumination. The chip and the BOE are both fabricated and experimentally demonstrated. Furthermore, the microflow cytometer is operated to detect fluorescence microbeads, whose experimental results are comparable to or even better than those of commercial instruments. It reveals the strong competitive power of the microflow cytometer.

## Figures and Tables

**Table 1:** Coefficient of variations (CVs) (%) of microbeads detected by the microflow cytometer and commercial instruments.

Flow Cytometer	Microflow Cytometer							BD Accuri C6			BD LSR Fortessa		
**Sample Flow Rate (μL/min)**	10	30	45	60	75	90	120	14	35	66	12	35	60
**3 μm Beads**	8.9	8.6	9.0	9.6	10.5	11.0	11.3	12.1	13.0	13.7	5.6	5.8	5.9
**5 μm Beads**	6.2	6.5	6.3	7.3	8.1	8.5	8.5	8.0	8.8	9.1	6.8	6.9	6.8
**7 μm Beads**	5.0	5.1	5.5	5.8	5.5	6.1	6.1	13.4	14.7	14.8	9.7	9.2	9.9

## Supplementary Materials

Figure S1: The focusing chip is made of five glass plates, of which the dimensions are 4 cm × 6 cm, and the microchannels are filled with a blue dye solution for visualization. Figure S2: The cross-sectional dimensions of the sample flow are quantified using image processing. Figure S3: Part of the BOE. Figure S4: Photo of the microflow cytometer system. Video S1: The amplitude fluctuation of fluorescence signal waveform increases with the sample flow rate.
