# Supplementary material for: A Microflow Cytometer with a Rectangular Quasi-Flat-Top Laser Spot
Source: Sensors (Basel). 2016 Sep 11;16(9):1474. doi: 10.3390/s16091474 (PMC5038752; doi:10.3390/s16091474)
Supplement: Supplementary file 1 [file sensors-16-01474-s001.pdf]

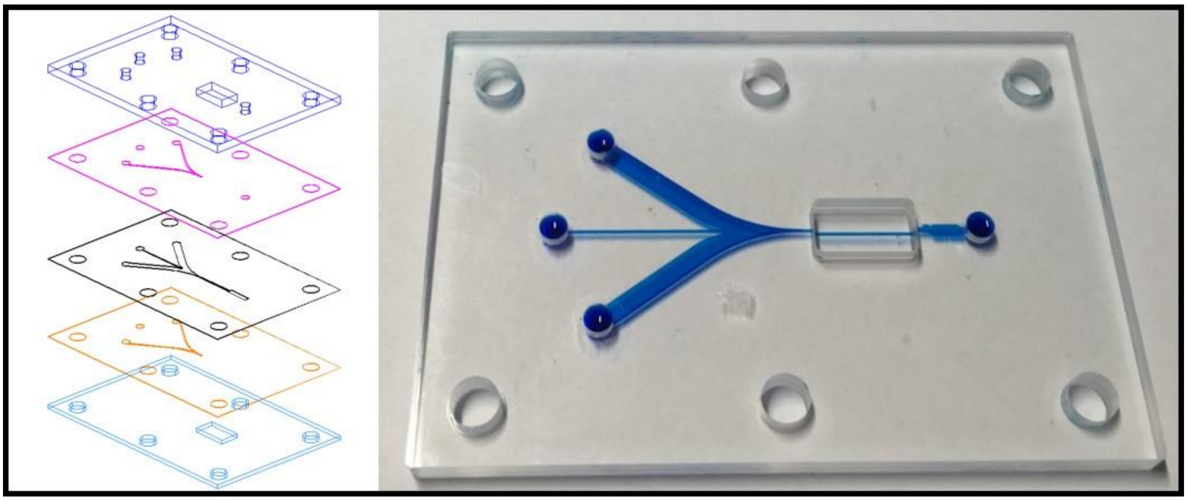

Figure S1. The focusing chip is made of five glass plates, of which the dimensions are 4 cm × 6 cm, and the microchannels are filled with a blue dye solution for visualization.

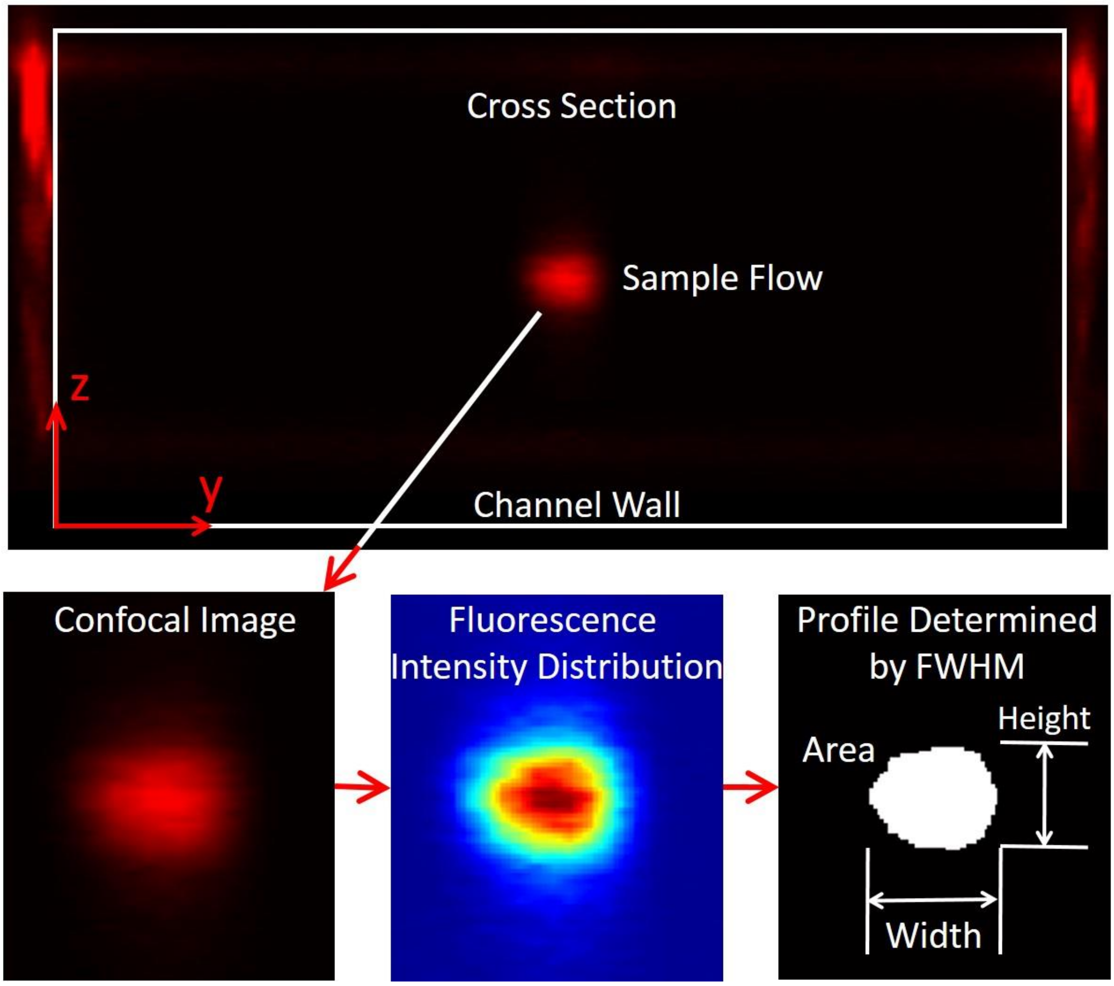

Figure S2. The cross-sectional dimensions of the sample flow are quantified using image processing.

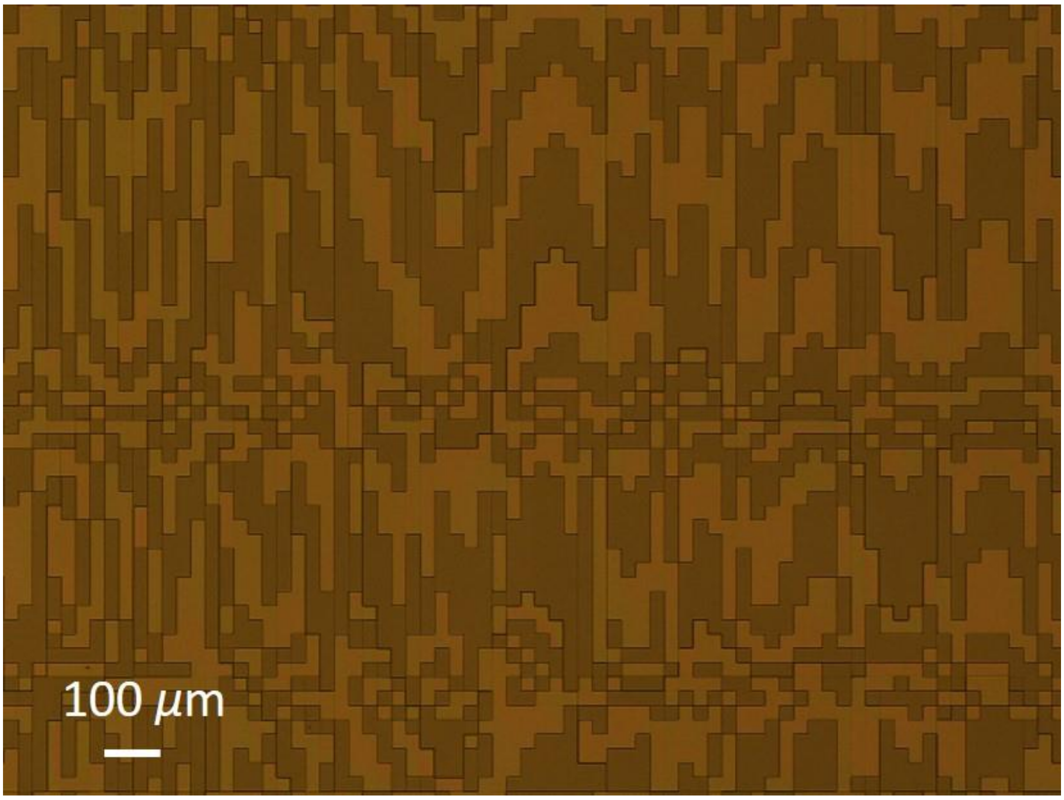

Figure S3. Part of the BOE.

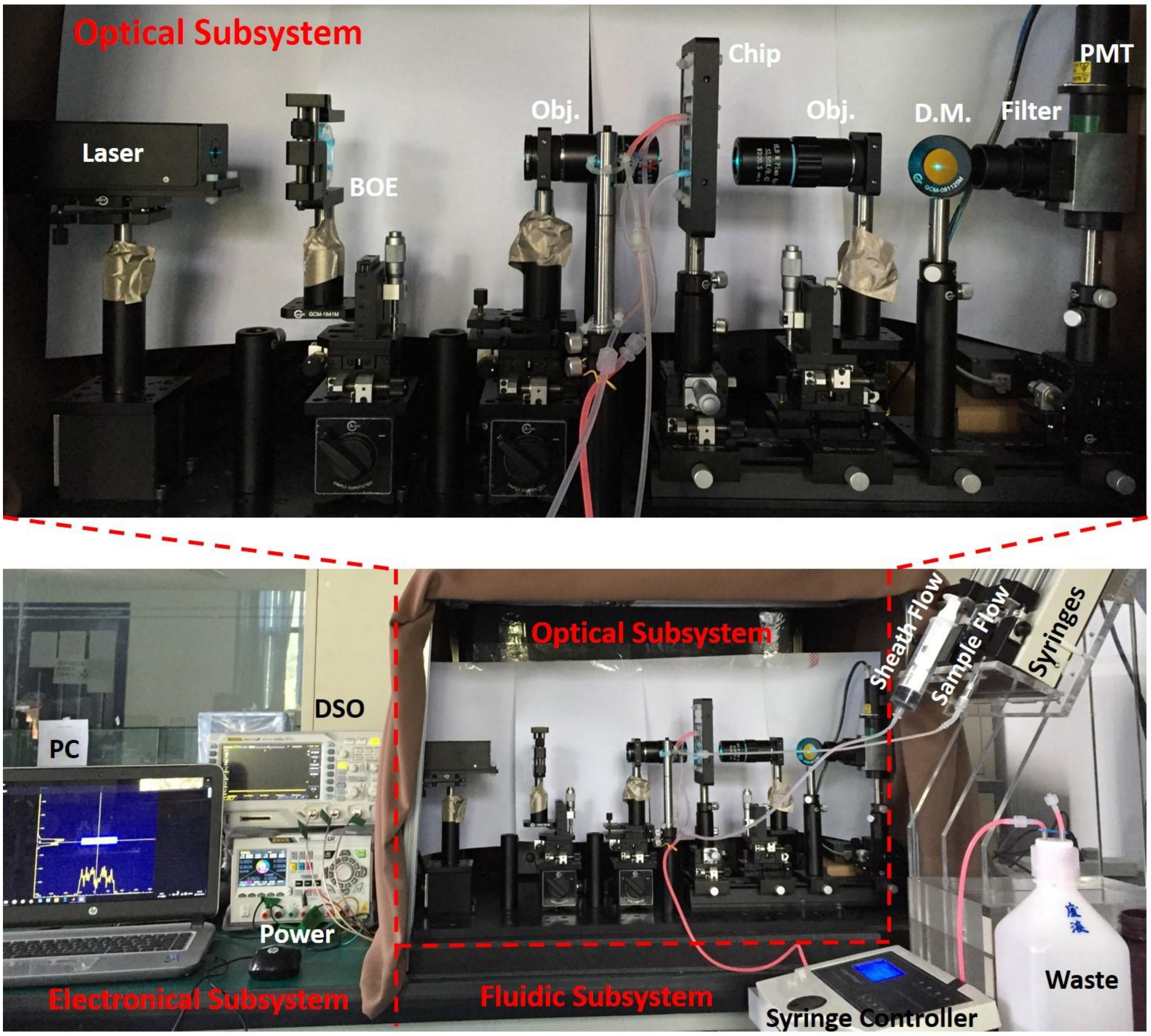

Figure S4. Photo of the microflow cytometer system.

Video S1. The amplitude fluctuation of fluorescence signal waveform increases with the sample flow rate.
